# Genome-wide association study of borderline personality disorder reveals genetic overlap with bipolar disorder, major depression and schizophrenia

**DOI:** 10.1038/tp.2017.115

**Published:** 2017-06-20

**Authors:** S H Witt, F Streit, M Jungkunz, J Frank, S Awasthi, C S Reinbold, J Treutlein, F Degenhardt, A J Forstner, S Heilmann-Heimbach, L Dietl, C E Schwarze, D Schendel, J Strohmaier, A Abdellaoui, R Adolfsson, T M Air, H Akil, M Alda, N Alliey-Rodriguez, O A Andreassen, G Babadjanova, N J Bass, M Bauer, B T Baune, F Bellivier, S Bergen, A Bethell, J M Biernacka, D H R Blackwood, M P Boks, D I Boomsma, A D Børglum, M Borrmann-Hassenbach, P Brennan, M Budde, H N Buttenschøn, E M Byrne, P Cervantes, T-K Clarke, N Craddock, C Cruceanu, D Curtis, P M Czerski, U Dannlowski, T Davis, E J C de Geus, A Di Florio, S Djurovic, E Domenici, H J Edenberg, B Etain, S B Fischer, L Forty, C Fraser, M A Frye, J M Fullerton, K Gade, E S Gershon, I Giegling, S D Gordon, K Gordon-Smith, H J Grabe, E K Green, T A Greenwood, M Grigoroiu-Serbanescu, J Guzman-Parra, L S Hall, M Hamshere, J Hauser, M Hautzinger, U Heilbronner, S Herms, S Hitturlingappa, P Hoffmann, P Holmans, J-J Hottenga, S Jamain, I Jones, L A Jones, A Juréus, R S Kahn, J Kammerer-Ciernioch, G Kirov, S Kittel-Schneider, S Kloiber, S V Knott, M Kogevinas, M Landén, M Leber, M Leboyer, Q S Li, J Lissowska, S Lucae, N G Martin, F Mayoral-Cleries, S L McElroy, A M McIntosh, J D McKay, A McQuillin, S E Medland, C M Middeldorp, Y Milaneschi, P B Mitchell, G W Montgomery, G Morken, O Mors, T W Mühleisen, B Müller-Myhsok, R M Myers, C M Nievergelt, J I Nurnberger, M C O'Donovan, L M O Loohuis, R Ophoff, L Oruc, M J Owen, S A Paciga, B W J H Penninx, A Perry, A Pfennig, J B Potash, M Preisig, A Reif, F Rivas, G A Rouleau, P R Schofield, T G Schulze, M Schwarz, L Scott, G C B Sinnamon, E A Stahl, J Strauss, G Turecki, S Van der Auwera, H Vedder, J B Vincent, G Willemsen, C C Witt, N R Wray, H S Xi, Bipolar Disorders Working Group of the Psychiatric Genomics Consortium, Major Depressive Disorder Working Group of the Psychiatric Genomics Consortium, Schizophrenia Working Group of the Psychiatric Genomics Consortium, A Tadic, N Dahmen, B H Schott, S Cichon, M M Nöthen, S Ripke, A Mobascher, D Rujescu, K Lieb, S Roepke, C Schmahl, M Bohus, M Rietschel

**Affiliations:** 1 Central Institute of Mental Health, Department of Genetic Epidemiology in Psychiatry, Medical Faculty Mannheim, Heidelberg University, Mannheim, Germany; 2 Central Institute of Mental Health, Clinic of Psychosomatic and Psychotherapeutic Medicine, Medical Faculty Mannheim, Heidelberg University, Mannheim, Germany; 3 Central Institute of Mental Health, Institute for Psychiatric and Psychosomatic Psychotherapy (IPPP)/Psychosomatic Medicine and Psychotherapy, Medical Faculty Mannheim, Heidelberg University, Mannheim, Germany; 4 Department of Psychiatry and Psychotherapy, Charité Universitätsmedizin Berlin, Campus Mitte, Berlin, Germany; 5 Human Genomics Research Group, Department of Biomedicine, University of Basel, Basel, Switzerland; 6 Institute of Human Genetics, University of Bonn, Bonn, Germany; 7 Life and Brain Center, Department of Genomics, University of Bonn, Bonn, Germany; 8 Department of Psychiatry (UPK), University of Basel, Basel, Switzerland; 9 Department of Psychiatry, Charité-Universitätsmedizin Berlin, Campus Benjamin Franklin, Berlin, Germany; 10 Department of Clinical Psychology and Psychotherapy, University of Heidelberg, Heidelberg, Germany; 11 Department of Biological Psychology, Vrije Universiteit Amsterdam, Amsterdam, The Netherlands; 12 Department of Clinical Sciences, Psychiatry, Umeå University, Umeå, Sweden; 13 Discipline of Psychiatry, University of Adelaide, Adelaide, SA, Australia; 14 Molecular and Behavioral Neuroscience Institute, University of Michigan, Ann Arbor, MI, USA; 15 Department of Psychiatry, Dalhousie University, Halifax, NS, Canada; 16 Department of Psychiatry and Behavioral Neuroscience, University of Chicago, Chicago, IL, USA; 17 Division Mental Health and Addiction, Oslo University Hospital, Oslo, Norway; 18 NORMENT, University of Oslo, Oslo, Norway; 19 Institute of Pulmonology, Russian State Medical University, Moscow, Russian Federation; 20 Division of Psychiatry, University College London, London, UK; 21 Department of Psychiatry and Psychotherapy, University Hospital Carl Gustav Carus, Dresden, Germany; 22 Inserm, U1144, AP-HP, GH Saint-Louis, Département de Psychiatrie et de Médecine Addictologique, Paris, France; 23 Department of Medical Epidemiology and Biostatistics, Karolinska Institutet, Stockholm, Sweden; 24 National Center for Mental Health, Cardiff University, Cardiff, UK; 25 Health Sciences Research, Mayo Clinic, Rochester, MN, USA; 26 Division of Psychiatry, University of Edinburgh, Edinburgh, UK; 27 Urain Center Rudolf Magnus, Department of Psychiatry, University Medical Center Utrecht, Utrecht, The Netherlands; 28 Department of Biomedicine, Aarhus University, Aarhus, Denmark; 29 iSEQ, Centre for Integrative Sequencing, Aarhus University, Aarhus, Denmark; 30 iPSYCH, The Lundbeck Foundation Initiative for Integrative Psychiatric Research, Aarhus, Denmark; 31 Medical and Quality Assurance, Clinics of Upper Bavaria, Munich, Germany; 32 Genetic Epidemiology Group, International Agency for Research on Cancer, Lyon, France; 33 Department of Psychiatry and Psychotherapy, University Medical Center Göttingen, Goettingen, Germany; 34 Medical Center of the University of Munich, Campus Innenstadt, Institute of Psychiatric Phenomics and Genomics (IPPG), Munich, Germany; 35 Translational Neuropsychiatry Unit, Department of Clinical Medicine, Aarhus University, Aarhus, Denmark; 36 Queensland Brain Institute, The University of Queensland, Brisbane, QLD, Australia; 37 Department of Psychiatry, McGill University, Montreal, QC, Canada; 38 Division of Psychological Medicine and Clinical Neurosciences, Cardiff University, Cardiff, UK; 39 Department of Translational Research in Psychiatry, Max Planck Institute of Psychiatry, Munich, Germany; 40 Centre for Psychiatry, Queen Mary University of London, London, UK; 41 UCL Genetics Institute, University College London, London, UK; 42 Laboratory of Psychiatric Genetics, Department of Psychiatry, Poznan University of Medical Sciences, Poznan, Poland; 43 Department of Psychiatry, University of Marburg, Marburg, Germany; 44 Department of Psychiatry, University of Münste, Münster, Germany; 45 Division of Psychological Medicine and Clinical Neurosciences, Cardiff University, Cardiff, UK; 46 Department of Medical Genetics, Oslo University Hospital Ullevål, Oslo, Norway; 47 NORMENT, KG Jebsen Centre for Psychosis Research, Department of Clinical Science, University of Bergen, Bergen, Norway; 48 Centre for Integrative Biology, Università degli Studi di Trento, Trento, Italy; 49 Indiana University School of Medicine, Department of Biochemistry and Molecular Biology, Indianapolis, IN, USA; 50 Indiana University School of Medicine, Department of Medical and Molecular Genetics, Indianapolis, IN, USA; 51 Faculté de Médecine, Université Paris Est, Créteil, France; 52 Department of Psychiatry and Psychology, Mayo Clinic, Rochester, MN, USA; 53 School of Medical Sciences, University of New South Wales, Sydney, NSW, Australia; 54 Neuroscience Research Australia, Sydney, NSW, Australia; 55 Department of Psychiatry, University of Halle, Halle, Germany; 56 Genetics and Computational Biology, QIMR Berghofer Medical Research Institute, Brisbane, QLD, Australia; 57 Department of Psychological Medicine, University of Worcester, Worcester, UK; 58 Department of Psychiatry and Psychotherapy, University Medicine Greifswald, Greifswald, Germany; 59 School of Biomedical and Healthcare Sciences, Plymouth University Peninsula Schools of Medicine and Dentistry, Plymouth, UK; 60 Department of Psychiatry, University of California San Diego, La Jolla, CA, USA; 61 Biometric Psychiatric Genetics Research Unit, Alexandru Obregia Clinical Psychiatric Hospital, Bucharest, Romania; 62 Mental Health Department, Biomedicine Institute, University Regional Hospital, Málaga, Spain; 63 Institute of Genetic Medicine, Newcastle University, Newcastle upon Tyne, UK; 64 Department of Psychology, Eberhard Karls Universität Tübingen, Tubingen, Germany; 65 Inserm U955, Psychiatrie Translationnelle, Créteil, France; 66 University Medical Center Utrecht, Division of Neuroscience, Department of Psychiatry, Utrecht, The Netherlands; 67 Center of Psychiatry Weinsberg, Weinsberg, Germany; 68 Department of Psychiatry, Psychosomatic Medicine and Psychotherapy, University Hospital Frankfurt am Main, Frankfurt am Main, Germany; 69 Centre for Addiction and Mental Health, Toronto, ON, Canada; 70 Department of Psychiatry, University of Toronto, Toronto, ON, Canada; 71 Max Planck Institute of Psychiatry, Munich, Germany; 72 Center for Research in Environmental Epidemiology (CREAL), Barcelona, Spain; 73 Institute of Neuroscience and Physiology, University of Gothenburg, Gothenburg, Sweden; 74 Clinic for Psychiatry and Psychotherapy, University Hospital Cologne, Cologne, Germany; 75 Inserm U955, Translational Psychiatry Laboratory, AP-HP, DHU PePSY, Department of Psychiatry, Université Paris Est, Créteil, France; 76 Janssen Research and Development, LLC, Neuroscience Therapeutic Area, Titusville, NJ, USA; 77 M. Sklodowska-Curie Cancer Center and Institute of Oncology, Cancer Epidemiology and Prevention, Warsaw, Poland; 78 School of Psychology, The University of Queensland, Brisbane, QLD, Australia; 79 Lindner Center of HOPE, Research Institute, Mason, OH, USA; 80 Centre for Cognitive Ageing and Cognitive Epidemiology, University of Edinburgh, Edinburgh, UK; 81 Genetic Cancer Susceptibility Group, International Agency for Research on Cancer, Lyon, France; 82 Division of Psychiatry, University College London, London, UK; 83 VU University Medical Center and GGZ inGeest, Department of Psychiatry, Amsterdam, The Netherlands; 84 School of Psychiatry, University of New South Wales, Sydney, NSW, Australia; 85 Black Dog Institute, Sydney, NSW, Australia; 86 Institute for Molecular Biology, University of Queensland, Brisbane, QLD, Australia; 87 Department of Neuroscience, Norwegian University of Science and Technology, Trondheim, Norway; 88 Department of Psychiatry, St Olavs University Hospital, Trondheim, Norway; 89 Risskov, Psychosis Research Unit, Aarhus University Hospital, Aarhus, Denmark; 90 iPSYCH, The Lundbeck Foundation Initiative for Integrative Psychiatric Research, Department of Clinical Medicine, Aarhus University, Aarhus, Denmark; 91 Research Center Juelich, Institute of Neuroscience and Medicine (INM-1), Juelich, Germany; 92 Division of Medical Genetics, University of Basel, Basel, Switzerland; 93 Munich Cluster for Systems Neurology (SyNergy), Munich, Germany; 94 University of Liverpool, Liverpool, UK; 95 HudsonAlpha Institute for Biotechnology, Huntsville, AL, USA; 96 Department of Psychiatry, Indiana University School of Medicine, Indianapolis, IN, USA; 97 MRC Centre for Neuropsychiatric Genetics and Genomics, Cardiff University, Cardiff, UK; 98 Center for Neurobehavioral Genetics, University of California Los Angeles, Los Angeles, CA, USA; 99 University Medical Center Utrecht, Division of Brain Research, Utrecht, The Netherlands; 100 Psychiatry Clinic, Clinical Center University of Sarajevo, Sarajevo, Bosnia-Herzegovina; 101 Pfizer Global Research and Development, Human Genetics and Computational Biomedicine, Groton, CT, USA; 102 Department of Psychiatry, University of Iowa, Iowa City, IA, USA; 103 Department of Psychiatry, Psychiatric University Hospital of Lausanne, Lausanne, Switzerland; 104 Department of Neurology and Neurosurgery, Faculty of Medicine, McGill University, Montreal, QC, Canada; 105 Montreal Neurological Institute and Hospital, Montreal, QC, Canada; 106 Department of Psychiatry and Behavioral Sciences, Johns Hopkins University, Baltimore, MD, USA; 107 NIMH Division of Intramural Research Programs, Human Genetics Branch, Bethesda, MD, USA; 108 Psychiatric Center Nordbaden, Wiesloch, Germany; 109 Center for Statistical Genetics, Department of Biostatistics, University of Michigan, Ann Arbor, MI, USA; 110 School of Medicine and Dentistry, James Cook University, Townsville, QLD, Australia; 111 Broad Institute of MIT and Harvard, Medical and Population Genetics, Cambridge, MA, USA; 112 Division of Psychiatric Genomics, Icahn School of Medicine at Mount Sinai, New York, NY, USA; 113 Centre for Addiction and Mental Health, Molecular Neuropsychiatry and Development Laboratory, Toronto, ON, Canada; 114 Department of Anaesthesiology and Operative Intensive Care, University Hospital Mannheim, Medical Faculty Mannheim/Heidelberg University, Mannheim, Germany; 115 Institute for Molecular Bioscience, The University of Queensland, Brisbane, QLD, Australia; 116 Pfizer Global Research and Development, Computational Sciences Center of Emphasis, Cambridge, MA, USA; 117 AGAPLESION Elisabethenstift gGmbh, Department of Psychiatry, Psychosomatics and Psychotherapy, Darmstadt, Germany; 118 University Medical Center, Department of Psychiatry and Psychotherapy, Mainz, Germany; 119 Leibniz Institute for Neurobiology, Magdeburg, Germany; 120 Department of Biomedicine, University of Basel, Basel, Switzerland; 121 Stanley Center for Psychiatric Research and Medical and Population Genetics Program, Broad Institute of MIT and Harvard, Cambridge, MA, USA; 122 Analytic and Translational Genetics Unit, Department of Medicine, Harvard Medical School, Massachusetts General Hospital, Boston, MA, USA

## Abstract

Borderline personality disorder (BOR) is determined by environmental and genetic factors, and characterized by affective instability and impulsivity, diagnostic symptoms also observed in manic phases of bipolar disorder (BIP). Up to 20% of BIP patients show comorbidity with BOR. This report describes the first case–control genome-wide association study (GWAS) of BOR, performed in one of the largest BOR patient samples worldwide. The focus of our analysis was (i) to detect genes and gene sets involved in BOR and (ii) to investigate the genetic overlap with BIP. As there is considerable genetic overlap between BIP, major depression (MDD) and schizophrenia (SCZ) and a high comorbidity of BOR and MDD, we also analyzed the genetic overlap of BOR with SCZ and MDD. GWAS, gene-based tests and gene-set analyses were performed in 998 BOR patients and 1545 controls. Linkage disequilibrium score regression was used to detect the genetic overlap between BOR and these disorders. Single marker analysis revealed no significant association after correction for multiple testing. Gene-based analysis yielded two significant genes: *DPYD* (*P*=4.42 × 10^−7^) and *PKP4* (*P*=8.67 × 10^−7^); and gene-set analysis yielded a significant finding for exocytosis (GO:0006887, *P*_FDR_=0.019; FDR, false discovery rate). Prior studies have implicated *DPYD*, *PKP4* and exocytosis in BIP and SCZ. The most notable finding of the present study was the genetic overlap of BOR with BIP (*r*_g_=0.28 [*P*=2.99 × 10^−3^]), SCZ (*r*_g_=0.34 [*P*=4.37 × 10^−5^]) and MDD (*r*_g_=0.57 [*P*=1.04 × 10^−3^]). We believe our study is the first to demonstrate that BOR overlaps with BIP, MDD and SCZ on the genetic level. Whether this is confined to transdiagnostic clinical symptoms should be examined in future studies.

## Introduction

Borderline personality disorder (BOR; for the sake of readability, we have decided to use the rather unconventional abbreviation ‘BOR’ for Borderline Personality Disorder and the abbreviation ‘BIP’ for Bipolar Disorder) is a complex neuropsychiatric disorder with a lifetime prevalence of around 3%.^1^ Untreated cases often have a chronic and severely debilitating clinical course.^1^ BOR affects up to 20% of all psychiatric inpatients, and is associated with high health-care utilization. BOR therefore represents a substantial socio-economic burden.^2, 3^

BOR is characterized by affective instability, emotional dysregulation and poor interpersonal functioning.^3^ Suicide rates in BOR range between 6 and 8%, and up to 90% of patients engage in non-suicidal self-injurious behavior.^4^ Other prototypical features include high-risk behaviors and impulsive aggression. Current theories view dysfunctions in emotion processing, social interaction and impulsivity as core psychological mechanisms of BOR.^5^

To date, genetic research into BOR has been limited. Available genetic studies have involved small samples and focused on candidate genes, while no genome-wide association study (GWAS) of BOR patients has yet been performed.^6^ However, Lubke *et al.*^7^ conducted a GWAS of borderline personality features using data from three cohorts comprising *n*=5802, *n*=1332 and *n*=1301 participants, respectively. Using the borderline subscale of the Personality Assessment Inventory (PAI-BOR), four borderline personality features (affect instability, identity problems, negative relations and self-harm) were assessed. The most promising signal in the combined analysis of two samples was for seven SNPs in the gene *SERINC5*, which encodes a protein involved in myelination. Two of the SNPs could be replicated in the third sample. Interestingly, here, the effect was highest for the affect instability items, that is, features that are key characteristics of manic phases of bipolar disorder (BIP).

Understanding of the pathogenesis of BOR remains limited. Both environmental and genetic factors are known to have a role in BOR etiology. Familial aggregation has been demonstrated,^8, 9^ and heritability estimates from twin studies range from 35 to 65%, with higher heritability estimates being obtained with self-ratings.^10, 11, 12^

The potential comorbidity between BOR and BIP is part of an ongoing debate. For example, Fornaro *et al.*^13^ report substantial comorbidity of ~20% with BIP, whereas Tsanas *et al.*^14^ find clear symptomatic differences between these two diagnostic groups. BOR displays an overlap of some symptoms with BIP, such as affective instability. In contrast, features such as dissociative symptoms, a feeling of chronic emptiness and identity disturbances are specific to BOR.^15^ To date, no twin or family study has generated conclusive results concerning a genetic overlap between the two disorders.^16, 17^ However, a twin study^18^ and a large-population-based study using polygenic risk score analyses^19^ indicate a genetic overlap between borderline personality features and neuroticism, an established risk factor for BIP and other psychiatric disorders.^20^

To the best of our knowledge, the present study represents the first case–control GWAS in BOR, and was performed in one of the largest BOR patient samples worldwide. Given the limited heritability and the expected complex genetic architecture of BOR, the sample is too small to generate significant results for single markers. Instead, the main aim of the investigation was to detect (i) genes and gene sets with a potential involvement in BOR; and (ii) potential genetic overlap with BIP. As a substantial overlap of common risk variants exists between BIP and schizophrenia (SCZ), and to a lesser extent between BIP and major depressive disorder (MDD), and as there is also a high comorbidity of BOR and MDD, a further aim of the study was to determine whether any observed genetic overlap between BOR and BIP, MDD and SCZ was driven by disorder-specific genetic factors using linkage disequilibrium (LD)-score regression and polygenic risk scores (PRS).

## Materials and methods

### Participants

The present sample comprised 1075 BOR patients and 1675 controls.^21^ All the participants provided written informed consent before inclusion. The study was approved by the respective local ethics committees.

The patients were recruited at the following German academic institutions: Department of Psychosomatic Medicine, Central Institute of Mental Health, Mannheim (*n*=350); Department of Psychiatry and Psychotherapy, University Medical Center Mainz (*n*=231); and the Department of Psychiatry, Charité, Campus Benjamin Franklin, Berlin (*n*=494). Inclusion criteria for patients were: age 16 to 65 years; Central European ancestry; and a lifetime DSM-IV diagnosis of BOR. The control sample comprised 1583 unscreened blood donors from Mannheim, and 92 subjects recruited by the University Medical Center Mainz.

### Clinical assessment

The diagnoses of BOR were assigned according to DSM-IV criteria and on the basis of structured clinical interviews. The diagnostic criteria for BOR were assessed using the German version of the IPDE^22^ or the SKID-II.^23^ All the diagnostic interviews were conducted by trained and experienced raters. BOR patients with a comorbid diagnosis of BIP or SCZ assessed with SKID-I^23^ were excluded.

### Genotyping

Automated genomic DNA extraction was performed using the chemagic Magnetic Separation Module I (Chemagen Biopolymer-Technologie, Baesweiler, Germany). Genotyping was performed using the Infinium PsychArray-24 Bead Chip (Illumina, San Diego, CA, USA).

### Quality control and imputation

A detailed description of the quality control and imputation procedures is provided elsewhere.^24^

Briefly, quality control parameters for the exclusion of subjects and single-nucleotide polymorphisms (SNPs) were: subject missingness >0.02; autosomal heterozygosity deviation (|Fhet|>0.2); SNP missingness >0.02; difference in SNP missingness between cases and controls >0.02; and SNP Hardy–Weinberg equilibrium (*P*<10^−6^ in controls; *P*<10^−10^ in cases).

Genotype imputation was performed using the pre-phasing/imputation stepwise approach in IMPUTE2/SHAPEIT (default parameters and a chunk size of 3 Mb),^25, 26^ using the 1000 Genomes Project reference panel (release ‘v3.macGT1’).^27^

Relatedness testing and population structure analysis were performed using a SNP subset that fulfilled strict quality criteria (INFO >0.8, missingness <1%, minor allele frequency >0.05), and which had been subjected to LD pruning (*r*^2^>0.02). This subset comprised 63 854 SNPs. In cryptically related subjects, one member of each pair (ðhat>0.2) was removed at random following the preferential retention of cases over controls. Principal components (PCs) were estimated from genotype data (see Supplementary Figures 1–6), and phenotype association was tested using logistic regression. The impact of the PCs on genome-wide test statistics was assessed using *λ*.

### Association analysis

Including the first four PCs as covariates, an additive logistic regression model was used to test single marker associations, as implemented in PLINK.^28^ The *P*-value threshold for genome-wide significance was set at 5 × 10^−8^.

### Gene-based analysis

To determine whether genes harbored an excess of variants with small *P*-values, a gene-based test was performed with MAGMA Version 1.04 (http://ctg.cncr.nl/software/magma)^29^ using genotyped markers only, filtered with a minor allele frequency >1% (*n*=284 220). This test uses summary data and takes LD between variants into account. SNPs within ±10 kb of the gene boundary were assigned to each gene. Obtained *P*-values were Bonferroni-corrected for the number of tested genes (*n*=17 755, *P*=2.8 × 10^−6^).

### Gene-set analysis

Gene-set-based analysis was implemented using genotyped markers only, filtered as above. As in the gene-based analysis, SNPs within ±10 kb of the gene boundary were assigned to each gene. Gene-set analyses were carried out using Gene Ontology (GO, http://software.broadinstitute.org/gsea/msigdb/) terms.

The discovery gene-set-based analysis was carried out using i-GSEA4GWASv2 (http://gsea4gwas-v2.psych.ac.cn/).^30^ The size of the gene sets was restricted to 20–200 genes, and the major histocompatibility complex region was excluded. In total, 674 gene sets were tested. The results were adjusted for multiple testing using false discovery rate (FDR). To validate the significant finding, the respective gene set was investigated with (i) GSA-SNP, using the *P*-value of the second-best SNP in each gene (https://gsa.muldas.org)^31^ and (ii) MAGMA using summary data and a nominal *P*-value threshold of *P*<0.05.

### LD-score regression

To investigate a possible genetic overlap between BOR and SCZ, BIP and MDD, LD-score regression was performed.^32^ Genetic correlations between BOR and (i) BIP, (ii) SCZ and (iii) MDD were calculated^33^ using the result files of the Psychiatric Genomics Consortium (PGC) meta-analyses for SCZ (33 640 cases and 43 456 controls),^34^ BIP (20 352 cases and 31 358 controls)^35^ and MDD (16 823 cases and 25 632 controls).^35^ There was no overlap in cases or controls of the present BOR GWAS sample with the PGC samples.

### Polygenic risk score

To determine the impact of polygenic risk on BOR and subgroups (that is, BOR with and without MDD), PRS were calculated for each subject based on the above-mentioned PGC data sets.

To obtain a highly informative SNP set with minimal statistical noise, the following were excluded: low frequency SNPs (minor allele frequency <0.1); low-quality variants (imputation INFO <0.9) and indels. Subsequently, these SNPs were clumped discarding markers within 500 kb of, and in high LD (*r*^2^⩾0.1) with, another more significant marker. From the major histocompatibility complex region, only one variant with the strongest significance was retained. PRS were calculated as described elsewhere.^36^ This involved *P*-value thresholds 5 × 10^−8^, 1 × 10^−6^, 1 × 10^−4^, 0.001, 0.01, 0.05, 0.1, 0.2, 0.5 and 1.0, and multiplication of the natural logarithm of the odds ratio of each variant by the imputation probability for the risk allele. The resulting values were then totaled. For each subject, this resulted in one PRS for SCZ, MDD and BIP for each *P*-value threshold.

In a first step, the association of the PRS for BIP, SCZ and MDD with BOR case–control status was analyzed using standard logistic regression and by including the four PCs as covariates. For each *P*-value threshold, the proportion of variance explained (Nagelkerke’s *R*^2^) in BOR case–control status was computed by comparison of a full model (covariates+PRS) score to a reduced model (covariates only).

For further exploratory analysis, the *P*<0.05 PRS for each disorder was selected (that is, including all markers that reached nominal significance in the training samples). To determine whether the different scores contribute independently to the case–control status, a regression including the PRS for MDD, SCZ and BIP and the four PCs was computed. In a secondary analysis, two further models were computed. These included the PRS for BIP and the PRS of either MDD or SCZ, while controlling for the four PCs.

Furthermore, PRS were analyzed by differentiating between controls, and patients with or without comorbid MDD. For each PRS, a linear model was computed using the PRS as a dependent variable, disease state as an independent variable and the four PCs as covariates. Differences between groups were assessed using *post hoc* tests (Bonferroni-corrected).

## Results

### Sample characteristics

Genetic quality control led to the exclusion of 207 subjects. Reasons for exclusion were: (i) insufficient data quality (low call rate), *n*=6; (ii) relatedness, *n*=63; and (iii) population outlier status, *n*=138. After quality control, the sample comprised 998 BOR cases (914 female/84 male) and 1545 controls (868 female/677 male). Mean age for cases was 29.58 years (range: 18–65 years, standard deviation (s.d.=8.64)). Mean age for controls was 44.19 years (range: 18–72 years, s.d.=13.24; details see Supplementary Table 1). Of the 998 cases, 666 had comorbid lifetime MDD, and 262 did not (data missing for 40 cases).

### Single marker analysis

**Figure 1:**
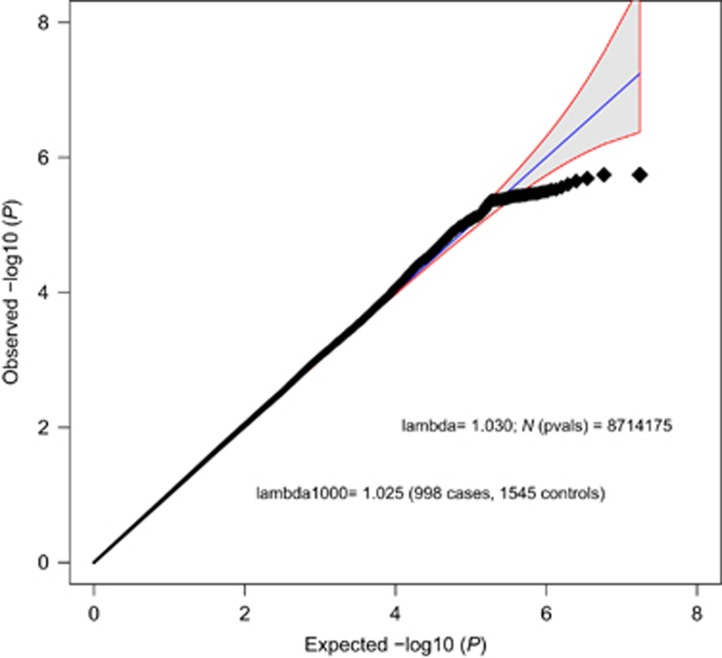
Quantile–Quantile plot. Quantile–Quantile plot of the case–control analysis (998 cases; 1545 controls) showing expected and observed –log10 *P*-values. The shaded region indicates the 95% confidence interval of expected *P*-values under the null hypothesis.

**Figure 2:**
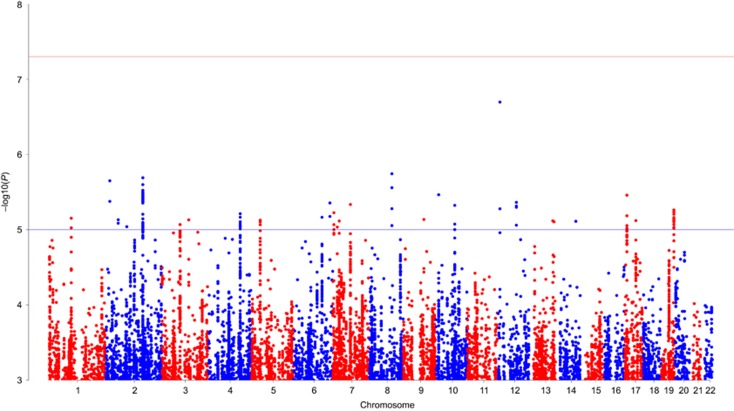
Manhattan plot showing association results. Manhattan plot of the case–control analysis (998 cases; 1545 controls). For each single-nucleotide polymorphism (SNP), the chromosomal position is shown on the *x* axis, and the –log10 *P*-value on the *y* axis. The red line indicates genome-wide significance (*P*<5 × 10^−8^) and the blue line indicates suggestive evidence for association (*P*<1 × 10^−5^).

A total of 10 736 316 single markers were included in the analysis. As expected for GWAS on a complex psychiatric disorder with the current sample size, the single marker analysis revealed no significant hit after correction for multiple testing (see Figures 1 and 2). The most significant marker was rs113507694 in *DPPA3* on chromosome 12 (*P*=2.01 × 10^−07^; odds ratio =0.35, minor allele frequency =0.03, INFO =0.59). Single markers with *P*<1 × 10^−5^ are listed in Supplementary Table 2.

### Gene-based analysis

In the gene-based analysis, a total of 17 755 genes were tested. Two genes showed significant association with BOR after correction for multiple testing: the gene coding for Plakophilin-4 on chromosome 2 (*PKP4*; *P*=8.24 × 10^−7^); and the gene coding for dihydropyrimidine dehydrogenase on chromosome 1 (*DPYD*, *P*=1.20 × 10^−6^). The most significant genes (*P*<5 × 10^−4^) are listed in Table 1. The top hit of the previous GWAS of borderline personality features, *SERINC5,* achieved nominal significance in the present study (*P*_uncorrected_=0.016).

### Gene-set analysis

Gene-set analysis with i-GSEA4GWASv2 revealed one significant gene set: exocytosis (GO: 0006887; *P*_FDR_=0.019). Of 25 genes in this gene set, 22 were mapped with variants and 15 showed nominally significant associations. Details on significant and nonsignificant genes in this gene set are provided in Supplementary Table 3. All gene sets with *P*_uncorrected_<0.01 are shown in Table 2. A technical replication analysis with GSA-SNP and MAGMA confirmed the gene-set exocytosis (GSA-SNP: *P*_uncorrected_=2.32 × 10^−4^; MAGMA: *P*_uncorrected_=0.056).

### LD-score regression

Significant genetic correlations with BOR were found for BIP (*r*_g_=0.28; s.e.=0.094; *P*=2.99 × 10^−3^), MDD (*r*_g_=0.57; s.e.=0.18; *P*=1.04 × 10^−3^) and SCZ (*r*_g_=0.34; s.e.=0.082; *P*=4.37 × 10^−5^). A meta-analytic comparison revealed no significant differences between the correlations (all *P*>0.13).

### Polygenic risk score

**Figure 3:**
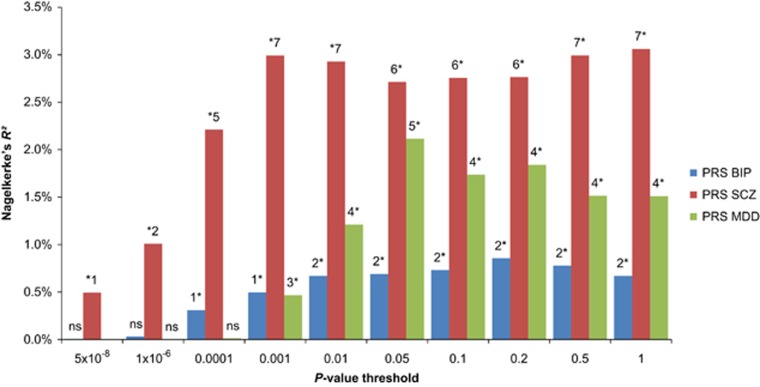
Polygenic risk score analysis. The proportion of variance explained in case–control status (*y* axis; Nagelkerke’s *R*^2^) by the PRS for BIP, SCZ and MDD is depicted for the different *P*-value cutoffs used in the calculation of the PRS. Principal components were included in the models to control for population stratification. 1*, *P*<0.05; 2*, *P*<0.001; 3*, *P*<1 × 10^−4^; 4*, *P*<1 × 10^−6^; 5*, *P*<1 × 10^−8^; 6*, *P*<1 × 10^−10^; 7*, *P*<1 × 10^−12^. BIP, bipolar disorder; MDD, major depressive disorder; NS, nonsignificant; PRS, polygenic risk score; SCZ, schizophrenia.

PRS analysis revealed significant associations with BOR for the PRS of BIP, MDD and SCZ. SCZ PRS were significant for all investigated thresholds. BIP and MDD scores were significant for all PRS that included SNPs with *P*-values higher than 0.0001 and 0.001, respectively (see Supplementary Table 4). The share of variance explained in BOR case–control status (Nagelkerke’s *R*^2^) by the respective PRS was up to 0.86% for BIP; up to 3.1% for SCZ; and up to 2.1% for MDD (see Figure 3 and Supplementary Table 4).

Simultaneous addition of the PRS for SCZ, BIP and MDD (threshold *P*<0.05) to the regression model explained 4.4% of the variance (Nagelkerke’s *R*^2^) in BOR case–control status. The PRS for SCZ and the PRS for MDD were significant predictors (*P*=9.78 × 10^−9^ and *P*=1.9 × 10^−7^, respectively). The PRS for BIP was not a significant predictor in this model (*P*=0.28).

A secondary analysis was then performed including (i) BIP PRS with MDD PRS and (ii) BIP PRS with SCZ PRS. Here, BIP PRS explained variance independently of MDD PRS (*P*=0.0067), but not of SCZ PRS (*P*=0.11).

**Figure 4:**
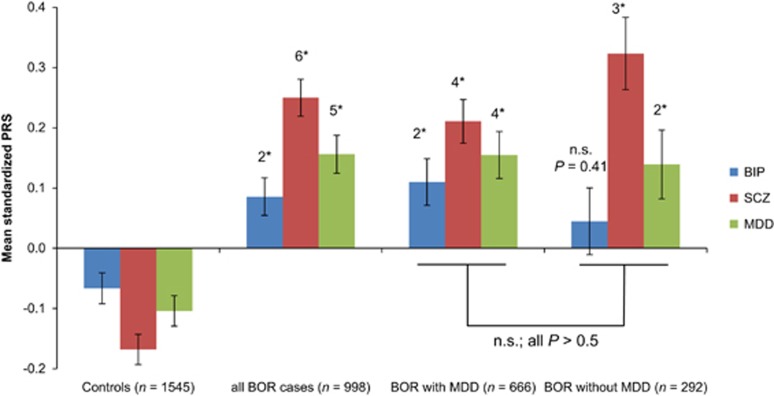
Polygenic risk score analysis in subgroups. Mean z-standardized PRS and standard error (s.e.) for BIP, SCZ and MDD are shown in the control group, all cases, and in cases with and without comorbid MDD. PRS with a *P*-value threshold of *P*=0.05 were selected for this comparison and principal components were included in the models to control for population stratification. The numbers at the top of each bar indicate the significance of the difference in the respective PRS in comparison with the control group. 1*, *P*<0.05; 2*, *P*<0.001; 3*, *P*<1 × 10^−4^; 4*, *P*<1 × 10^−6^; 5*, *P*<1 × 10^−8^; 6*, *P*<1 × 10^−10^; 7*, *P*<1 × 10^−12^. BIP, bipolar disorder; BOR, borderline personality disorder; MDD, major depressive disorder; NS, nonsignificant; PRS, polygenic risk score; SCZ, schizophrenia.

Differentiation between cases with and without comorbid MDD and controls revealed significant effects of BOR diagnosis on PRS for BIP, SCZ and MDD (all *P*<0.001, see Figure 4). *Post hoc* analyses revealed no differences in PRS for the BIP, SCZ or MDD PRS of the BOR subgroup with comorbid MDD compared with the BOR subgroup without MDD (all *P*>0.5).

Compared with controls, PRS for SCZ and MDD were significantly increased in the BOR subgroups with and without comorbid MDD (all *P*<0.001). The PRS for BIP only showed a significant difference to controls in the BOR subgroup with comorbid MDD (*P*<0.001, see Figure 4).

## Discussion

The present study is the first case–control GWAS of BOR. As expected, no genome-wide significant association was found for any single marker. In the gene-based test, however, two genes achieved genome-wide significance: dihydropyrimidine dehydrogenase (*DPYD*) and Plakophilin-4 (*PKP4*). *DPYD* encodes a pyrimidine catabolic enzyme, which is the initial and rate-limiting factor in the pathway of uracil and thymidine catabolism. Genetic deficiency of this enzyme results in an error in pyrimidine metabolism.^37^ This is associated with thymine–uraciluria and an increased risk of toxicity in cancer patients receiving 5-fluorouracil chemotherapy (http://www.ncbi.nlm.nih.gov/gene/1806). Recent PGC meta-analyses revealed an association between *DPYD* and SCZ and BIP.^34, 38, 39^ *DPYD* contains a binding site for the micro-RNA miR-137, which has previously been associated with schizophrenia,^40^ and a previous exome-sequencing study reported two putative functional *de novo* variants in *DPYD* in cases with SCZ.^41^ *PKP4* is involved in the regulation of cell adhesion and cytoskeletal organization.^42^ In pathway analyses of PGC GWAS data, cell adhesion was associated with BIP,^43^ and SCZ,^44^ whereas cell junction was implicated in MDD, as well as in an integrative pathway analysis of all three disorders.^45^

*SERINC5,* which was the top hit of the previous GWAS of Borderline personality features,^7^ achieved nominal significance in the present study. The protein SERINC5 incorporates serine into newly forming membrane lipids, and is enriched in myelin in the brain.^46^ Previous research suggests that decreased myelination is associated with a reduced capacity for social interaction.^7, 47^

The gene-set analyses yielded significant results for exocytosis. In neuronal synapses, exocytosis is triggered by an influx of calcium and critically underlies synaptic signaling. Dysregulated neuronal signaling and exocytosis are core features of neurodevelopmental psychiatric disorders such as the autism spectrum disorders and intellectual disability.^48, 49^ Moreover, recent findings from large meta-analyses have implicated dysregulated neuronal signaling and exocytosis in the molecular mechanisms of BIP, SCZ and MDD.^48, 50, 51^ These processes may now represent promising starting points for further research into BOR.

The most interesting finding of this study is that BOR showed a genetic overlap with BIP, SCZ and MDD. Notably, BIP did not show a higher correlation with BOR (*r*_g_=0.28) than SCZ (*r*_g_=0.34) or MDD (*r*_g_=0.57). In view of the present sample size, these values must be viewed with caution. A more accurate estimation of these correlations will require calculations in larger cohorts.

Although comorbid BIP was excluded in the present BOR patients, the possibility that the observed genetic overlap between BOR and BIP was at least partly attributable to misdiagnosis cannot be excluded. However, an alternative explanation appears more likely, that is, that disorders currently categorized as BOR and BIP share a common genetic background, and they also do so with SCZ and MDD. This hypothesis is supported by the present observation of a genetic overlap between BOR and SCZ, two disorders that are rarely misdiagnosed by psychiatrists, despite the presence of common psychotic symptoms.

An explanation could also be that the genetic commonality between BOR and BIP, SCZ, and MDD might be due to a common effect of MDD. Prior to the introduction of DSM-IV, a history of MDD was required for a diagnosis of BIP, and MDD has a high prevalence in patients with SCZ (25-85%).^52, 53^ Therefore, the MDD genetic risk variants that are common to BOR, BIP, and SCZ may be responsible for the observed overlap. For this reason, we conducted two further analyses. First, we compared PRS of BIP, SCZ and MDD in subsamples of BOR patients with (~60%) and without comorbid MDD. Here, no differences in any of the PRS were found. Second, we performed a joint analysis of PRS of BIP, SCZ and MDD in a logistic regression analysis in BOR patients vs controls. Here, no differences were found in any of the PRS. Second, we performed a joint analysis of the PRS of BIP, SCZ and MDD in a logistic regression analysis in BOR patients vs controls. Here, both the SCZ and the MDD risk score explained variance in BOR case–control status independently. Secondary analysis revealed that the BIP risk score explained variance independently of the MDD risk score but not of the SCZ risk score. These results indicate that comorbidity with MDD does not explain the genetic overlap between BOR and BIP, SCZ and MDD. However, the training sets differ in terms of their power to detect underlying risk variants, and therefore the derived PRS differ in terms of the variance they can explain.

It must be noted, that in the PGC-BIP, -SCZ and -MDD samples, controls are partly overlapping. However, it is unlikely that this drives the genetic correlation of BOR with those disorders as the overlap of controls in these samples is rather small (under 10%).^54^ Also, the joint logistic regression analysis demonstrated that polygenic risk for SCZ and MDD contributed independently to the BOR risk (see above).

The present study had several limitations. First, despite being one of the largest BOR samples available worldwide, the sample size was small in terms of the estimation of heritability. Replication of the present results is warranted in larger, independent cohorts. This should include the investigation of non-European samples. Second, no information was available on the presence of common clinical features such as psychotic symptoms and affect instability. This precluded detailed analysis of the identified genetic overlap. Future studies in larger cohorts should also investigate more detailed phenotypes, including comorbid axis I and axis II disorders, such as addiction and personality disorders, respectively. Third, the observation that psychiatric patients often establish non-random relationships with persons affected by the same or another psychiatric disorder,^55^ and therefore have offspring with a higher genetic risk for psychiatric disorders, might contribute to the observed genetic correlation of BOR with BIP, SCZ and MDD. However, the LD-score method does not investigate the impact of assortative mating.^32^ Therefore, assessment of the degree to which this phenomenon may have influenced the genetic correlation estimates was beyond the scope of the present study.

Despite these limitations, the results indicate that neither comorbidity with MDD nor risk variants that are exclusive to MDD explain the genetic overlap between BOR and BIP, SCZ and MDD. Future investigations of larger data sets for BOR and other psychiatric disorders are warranted to refine the analysis of shared and specific genetic risk.

Future studies are warranted to delineate the communalities and specificities of the respective disorders.

## Conclusion

In summary, the present study is the first GWAS of patients diagnosed with BOR. The results suggest promising novel genes and a novel pathway for BOR, and demonstrate that, rather than being a discrete entity, BOR has an etiological overlap with the major psychoses. The genetic overlap with BIP is consistent with the observation that some diagnostic criteria for BOR overlap with those for BIP. The overlap between BOR and SCZ and MDD is consistent with previous observations of genetic overlap of other psychiatric disorders.^56^ Given that BOR patients display specific clinical symptoms not observed in patients with other psychiatric disorders, knowledge of shared and non-shared genetic and clinical features will be important for the development of personalized treatment approaches.

## Figures and Tables

**Table 1:** Results of the gene-based analysis using MAGMA

*GENE*	*CHR*	*START*	*STOP*	N*_SNPS_*	N*_PARAM_*	Z*_STAT_*	P
***PKP4***	2	159303476	159547941	21	13	4.7924	8.24 × 10^−7^
***DPYD***	1	97533299	98396615	105	68	4.7162	1.20 × 10^−6^
GRAMD1B	11	123315191	123508478	34	28	3.8856	5.10 × 10^−5^
STX8	17	9143788	9489275	38	33	3.7984	7.28 × 10^−5^
BMP2	20	6738745	6770910	7	6	3.588	1.67 × 10^−4^
*TRAF3IP1*	2	239219185	239319541	11	8	3.5389	2.01 × 10^−4^
ZP3	7	76016841	76081388	9	7	3.5037	2.29 × 10^−4^
*PINX1*	8	10612473	10707394	19	11	3.5034	2.30 × 10^−4^
GTF3C4	9	135535728	135575471	4	4	3.4851	2.46 × 10^−4^
DNAH1	3	52340335	52444513	11	8	3.4543	2.76 × 10^−4^
YKT6	7	44230577	44263893	6	3	3.3841	3.57 × 10^−4^
CCSER1	4	91038684	92533370	111	78	3.3804	3.62 × 10^−4^
LRRC59	17	48448594	48484914	8	6	3.3716	3.74 × 10^−4^
TMEM71	8	133712191	133782914	9	8	3.3668	3.80 × 10^−4^
BAP1	3	52425020	52454121	3	3	3.345	4.11 × 10^−4^
AQR	15	35138552	35271995	8	6	3.3299	4.34 × 10^−4^
FGFR1	8	38258656	38336352	12	10	3.3162	4.56 × 10^−4^

Abbreviations: CHR, chromosome; *N*_PARAM_, number of parameters used in the model; *N*_SNPS_, number of single-nucleotide polymorphisms; *P*, *P*-value of gene; *Z*_STAT_, *z*-value of the gene.

Most significant genes (*P*<5 × 10^−4^) in the gene-based analysis and their chromosomal position. Genes in bold font were significant after correction for multiple testing.

**Table 2:** Results of the gene-set analysis

*Gene-set name*	*Number of genes*	P*-value*	*FDR* P*-value*
**GO: EXOCYTOSIS**	**25**	**0.001**	**0.019**
GO: RESPONSE TO ORGANIC SUBSTANCE	30	0.002	0.173
GO: BRAIN DEVELOPMENT	51	0.003	0.888
GO: HORMONE METABOLIC PROCESS	30	0.003	0.511
GO: PROTEIN C TERMINUS BINDING	73	0.003	0.536
GO: LYSOSOME	53	0.007	0.785
GO: LYTIC VACUOLE	53	0.007	0.785
GO: MULTI-ORGANISM PROCESS	143	0.007	0.920

Abbreviations: FDR, false discovery rate; GO, Gene Ontology; *P*-value, gene-set *P*-value.

Most significant gene sets (uncorrected *P*<0.01) in the gene-set analysis with i-GSEA4GWASv2 are listed. Gene sets in bold font were significant after correction for multiple testing.
