# Supplementary material for: Genome-wide association study of borderline personality disorder reveals genetic overlap with bipolar disorder, major depression and schizophrenia
Source: Transl Psychiatry. 2017 Jun 20;7(6):e1155–. doi: 10.1038/tp.2017.115 (PMC5537640; doi:10.1038/tp.2017.115)
Supplement: Supplementary Material [file tp2017115x1.doc]

Supplementary Information for *Genome-wide association study of Borderline Personality Disorder reveals genetic overlap with the Major Psychoses*

Table of Content

[Figures 2](#__RefHeading___Toc473815019)

[Supplementary Figure 1: Scatter plot of principal components 1 and 2 2](#__RefHeading___Toc473815020)

[Supplementary Figure 2: Scatter plot of principal components 1 and 3 2](#__RefHeading___Toc473815021)

[Supplementary Figure 3: Scatter plot of principal components 2 and 3 3](#__RefHeading___Toc473815022)

[Supplementary Figure 4: Scatter plot of principal components 2 and 4 3](#__RefHeading___Toc473815023)

[Supplementary Figure 5: Scatter plot of principal components 3 and 4 4](#__RefHeading___Toc473815024)

[Supplementary Figure 6: Scatter plot of principal components 5 and 6 4](#__RefHeading___Toc473815025)

[Supplementary Figure 7: Region plot of PKP4 5](#__RefHeading___Toc473815026)

[Supplementary Figure 8: Region plot of DPYD 5](#__RefHeading___Toc473815027)

[Tables 6](#__RefHeading___Toc473815028)

[Supplementary Table 1: Demographic and clinical information on the final sample 6](#__RefHeading___Toc473815029)

[Supplementary Table 2: Association results for single markers 7](#__RefHeading___Toc473815030)

[Supplementary Table 3: Overview of Gene Set Excytosis (GO: 0006887) 13](#__RefHeading___Toc473815031)

[Supplementary Table 4: Association of polygenic risk scores for Bipolar Disorder (BIP), Schizophrenia (SCZ), and Major Depressive Disorder (MDD) with Borderline Personality Disorder case control status 14](#__RefHeading___Toc473815032)

[Consortium Memberships 15](#__RefHeading___Toc473815033)

[Bipolar Disorders Working Group of the Psychiatric Genomics Consortium (Bipolar disorder) 15](#__RefHeading___Toc473815034)

[Major Depressive Disorder Working Group of the Psychiatric Genomics Consortium (Major depressive disorder) 16](#__RefHeading___Toc473815035)

# Figures

## Supplementary Figure 1: Scatter plot of principal components 1 and 2

## Supplementary Figure 2: Scatter plot of principal components 1 and 3

## Supplementary Figure 3: Scatter plot of principal components 2 and 3

## Supplementary Figure 4: Scatter plot of principal components 2 and 4

## Supplementary Figure 5: Scatter plot of principal components 3 and 4

## Supplementary Figure 6: Scatter plot of principal components 5 and 6

## Supplementary Figure 7: Region plot of PKP4

## Supplementary Figure 8: Region plot of DPYD

# Tables

## Supplementary Table 1: Demographic and clinical information on the final sample

|  | **Cases** | **Controls** |
| --- | --- | --- |
| **N** | 998 | 1,545 |
| **Sex (female/male)** | 914 / 84 | 868 / 677 |
| **Age in years (SD)** | 29.58 (8.64) | 44.19 (13.24) |
|  |  |  |
| **Comorbidity** |  |  |
| **Depression** |  |  |
| **(yes / no / missing)** | 666 / 262 / 40 | - |
|  |  |  |
| **Alcohol dependency** |  |  |
| **(yes / no / missing)** | 163/ 781 / 54 | - |

## Supplementary Table 2: Association results for single markers

Markers are reported with p<1x10^-5^, and sorted according to chromosomal position. CHR = chromosome, BP = base pair position, A = allele, FRQ = frequency, SNP = single nucleotide polymorphism, OR = odds ratio, INFO = imputation info score, SE = standard error, GT = genotyped, IMP = imputed

| ***CHR*** | ***SNP*** | ***BP*** | ***Gene*** | ***A1*** | ***A2*** | ***FRQ cases*** | ***FRQ controls*** | ***INFO*** | ***OR*** | ***SE*** | ***P*** | ***GT*** |
| --- | --- | --- | --- | --- | --- | --- | --- | --- | --- | --- | --- | --- |
| **1** | rs187785463 | 98152125 | DPYD | A | G | 0.0531 | 0.0739 | 0.752 | 0.4935 | 0.1572 | 7.05E-06 | IMP |
| **1** | rs6683957 | 98200719 | DPYD | A | G | 0.078 | 0.1128 | 1.002 | 0.6117 | 0.111 | 9.46E-06 | IMP |
| **2** | rs57726666 | 16203326 | GACAT3 | A | G | 0.9407 | 0.9125 | 0.912 | 1.8855 | 0.1341 | 2.24E-06 | IMP |
| **2** | rs78068563 | 16208690 | GACAT3 | A | G | 0.9397 | 0.9127 | 0.9227 | 1.8395 | 0.1324 | 4.19E-06 | IMP |
| **2** | rs115689122 | 52606079 | AC087073.1 | A | T | 0.016 | 0.0054 | 0.7809 | 6.1189 | 0.4062 | 8.20E-06 | IMP |
| **2** | rs62127626 | 52719291 | AC139712.4 | A | C | 0.0163 | 0.0057 | 0.7744 | 5.9639 | 0.3984 | 7.38E-06 | IMP |
| **2** | rs150592717 | 89936117 |  | T | C | 0.8884 | 0.869 | 0.3247 | 2.2187 | 0.1796 | 9.14E-06 | IMP |
| **2** | rs4664975 | 159418438 | PKP4 | A | C | 0.4677 | 0.5152 | 0.8943 | 0.7399 | 0.0678 | 8.77E-06 | IMP |
| **2** | rs3771608 | 159419739 | PKP4 | A | C | 0.5405 | 0.4847 | 0.9831 | 1.345 | 0.0646 | 4.42E-06 | IMP |
| **2** | rs12052933 | 159420140 | PKP4 | A | G | 0.54 | 0.4841 | 0.9852 | 1.3451 | 0.0645 | 4.29E-06 | IMP |
| **2** | rs10174340 | 159421412 | PKP4 | A | T | 0.54 | 0.4841 | 0.9856 | 1.3451 | 0.0645 | 4.27E-06 | IMP |
| **2** | rs3771609 | 159422239 | PKP4 | T | C | 0.46 | 0.516 | 0.9859 | 0.7434 | 0.0645 | 4.25E-06 | IMP |
| **2** | rs3771610 | 159422317 | PKP4 | A | G | 0.54 | 0.484 | 0.9859 | 1.3451 | 0.0645 | 4.25E-06 | IMP |
| **2** | rs10187426 | 159425104 | PKP4 | A | G | 0.5399 | 0.4839 | 0.9869 | 1.3451 | 0.0644 | 4.21E-06 | IMP |
| **2** | rs7577672 | 159429019 | PKP4 | A | G | 0.4748 | 0.5324 | 0.9886 | 0.7523 | 0.0643 | 9.56E-06 | IMP |
| **2** | rs3771614 | 159430579 | PKP4 | T | G | 0.5318 | 0.4731 | 0.9904 | 1.3443 | 0.0643 | 4.26E-06 | IMP |
| **2** | rs3771616 | 159430991 | PKP4 | A | G | 0.5318 | 0.4731 | 0.9903 | 1.3444 | 0.0643 | 4.23E-06 | IMP |
| **2** | rs3821291 | 159431031 | PKP4 | A | G | 0.5318 | 0.4731 | 0.9903 | 1.3444 | 0.0643 | 4.23E-06 | IMP |
| **2** | rs12473797 | 159431663 | PKP4 | T | C | 0.5319 | 0.4732 | 0.9898 | 1.3449 | 0.0644 | 4.14E-06 | IMP |
| **2** | rs3755408 | 159431842 | PKP4 | T | C | 0.5318 | 0.4731 | 0.9903 | 1.3445 | 0.0643 | 4.21E-06 | IMP |
| **2** | chr2_159432313_D | 159432313 | PKP4 | I2 | D | 0.5496 | 0.4914 | 0.9516 | 1.3537 | 0.0658 | 4.13E-06 | IMP |
| **2** | rs2356189 | 159434500 | PKP4 | A | G | 0.5402 | 0.4839 | 0.9869 | 1.3482 | 0.0644 | 3.55E-06 | IMP |
| **2** | rs3755413 | 159436702 | PKP4 | A | G | 0.528 | 0.4703 | 0.9825 | 1.3387 | 0.0646 | 6.23E-06 | IMP |
| **2** | rs3771620 | 159438129 | PKP4 | T | C | 0.4591 | 0.5157 | 0.9808 | 0.7375 | 0.0647 | 2.52E-06 | IMP |
| **2** | rs4664979 | 159439898 | PKP4 | T | C | 0.4597 | 0.5161 | 0.9868 | 0.7414 | 0.0644 | 3.44E-06 | IMP |
| **2** | rs3771627 | 159443266 | PKP4 | T | C | 0.4596 | 0.5161 | 0.9865 | 0.741 | 0.0645 | 3.31E-06 | IMP |
| **2** | rs2108215 | 159446818 | PKP4 | T | G | 0.4674 | 0.5269 | 0.9909 | 0.7404 | 0.0643 | 3.01E-06 | GT |
| **2** | rs11891131 | 159447509 | PKP4 | T | C | 0.532 | 0.4731 | 0.9898 | 1.347 | 0.0644 | 3.71E-06 | IMP |
| **2** | rs1465236 | 159448238 | PKP4 | A | G | 0.4682 | 0.527 | 0.9894 | 0.7424 | 0.0644 | 3.71E-06 | IMP |
| **2** | rs999232 | 159448306 | PKP4 | C | G | 0.468 | 0.5269 | 0.9899 | 0.7422 | 0.0644 | 3.62E-06 | IMP |
| **2** | rs3771631 | 159448926 | PKP4 | A | C | 0.468 | 0.5269 | 0.9898 | 0.7422 | 0.0644 | 3.62E-06 | IMP |
| **2** | rs3771632 | 159448946 | PKP4 | C | G | 0.5341 | 0.4763 | 0.9806 | 1.3485 | 0.0646 | 3.75E-06 | IMP |
| **2** | rs2051946 | 159454789 | PKP4 | T | G | 0.532 | 0.4732 | 0.9886 | 1.3474 | 0.0644 | 3.66E-06 | IMP |
| **2** | rs2051947 | 159459863 | PKP4 | A | G | 0.5321 | 0.4733 | 0.9884 | 1.3472 | 0.0644 | 3.71E-06 | IMP |
| **2** | rs12694965 | 159460218 | PKP4 | T | C | 0.4594 | 0.5159 | 0.9836 | 0.7396 | 0.0646 | 3.00E-06 | IMP |
| **2** | rs10191923 | 159460807 | PKP4 | T | C | 0.5321 | 0.4735 | 0.9882 | 1.3467 | 0.0644 | 3.83E-06 | IMP |
| **2** | rs10191934 | 159460832 | PKP4 | T | G | 0.5322 | 0.4736 | 0.9877 | 1.3461 | 0.0644 | 3.97E-06 | IMP |
| **2** | rs10191939 | 159460846 | PKP4 | A | T | 0.4679 | 0.5267 | 0.9885 | 0.7423 | 0.0644 | 3.72E-06 | IMP |
| **2** | chr2_159465312_I | 159465312 | PKP4 | I5 | D | 0.4498 | 0.5053 | 0.9315 | 0.7371 | 0.0663 | 4.28E-06 | IMP |
| **2** | rs3771643 | 159466149 | PKP4 | T | C | 0.532 | 0.4731 | 0.9889 | 1.3483 | 0.0644 | 3.46E-06 | IMP |
| **2** | rs3771647 | 159469420 | PKP4 | T | C | 0.5321 | 0.4735 | 0.9893 | 1.3459 | 0.0644 | 3.93E-06 | IMP |
| **2** | rs7607589 | 159469920 | PKP4 | T | C | 0.4594 | 0.5159 | 0.9845 | 0.7404 | 0.0645 | 3.18E-06 | GT |
| **2** | chr2_159476445_D | 159476445 | PKP4 | I13 | D | 0.5489 | 0.4908 | 0.9403 | 1.3685 | 0.0661 | 2.04E-06 | IMP |
| **2** | rs10180170 | 159476635 | PKP4 | T | C | 0.532 | 0.4735 | 0.9892 | 1.3459 | 0.0644 | 3.95E-06 | IMP |
| **2** | rs10180277 | 159476732 | PKP4 | T | C | 0.5325 | 0.4735 | 0.99 | 1.3493 | 0.0644 | 3.23E-06 | IMP |
| **2** | rs2883873 | 159486346 | PKP4 | T | C | 0.4596 | 0.5149 | 0.9828 | 0.742 | 0.0646 | 3.84E-06 | IMP |
| **2** | rs2528638 | 159500560 | PKP4 | A | G | 0.4597 | 0.5145 | 0.98 | 0.7423 | 0.0647 | 4.08E-06 | IMP |
| **2** | rs2711070 | 159502533 | PKP4 | C | G | 0.4598 | 0.5145 | 0.9798 | 0.7428 | 0.0647 | 4.27E-06 | IMP |
| **2** | rs929390 | 159503016 | PKP4 | A | G | 0.4598 | 0.5145 | 0.9798 | 0.7428 | 0.0647 | 4.28E-06 | IMP |
| **2** | rs2711068 | 159503757 | PKP4 | A | G | 0.4561 | 0.5082 | 0.9498 | 0.7482 | 0.0657 | 9.98E-06 | IMP |
| **2** | rs2711067 | 159503758 | PKP4 | C | G | 0.4558 | 0.5082 | 0.9496 | 0.7476 | 0.0657 | 9.41E-06 | IMP |
| **2** | chr2_159505404_D | 159505404 | PKP4 | I7 | D | 0.5375 | 0.4842 | 0.9621 | 1.3353 | 0.0652 | 9.12E-06 | IMP |
| **2** | rs2711062 | 159511527 | PKP4 | A | T | 0.537 | 0.4807 | 0.967 | 1.3501 | 0.0652 | 4.09E-06 | IMP |
| **2** | chr2_159523551_D | 159523551 | PKP4 | I2 | D | 0.5395 | 0.4867 | 0.969 | 1.3392 | 0.0651 | 7.17E-06 | IMP |
| **2** | rs2711072 | 159523803 | PKP4 | C | G | 0.5399 | 0.4876 | 0.9688 | 1.335 | 0.0651 | 9.00E-06 | IMP |
| **2** | rs2711073 | 159525516 | PKP4 | A | T | 0.4611 | 0.5138 | 0.9713 | 0.7487 | 0.065 | 8.46E-06 | IMP |
| **2** | rs2528590 | 159527845 | PKP4 | A | T | 0.4586 | 0.5105 | 0.9641 | 0.7477 | 0.0653 | 8.33E-06 | IMP |
| **2** | rs2528591 | 159527936 | PKP4 | T | C | 0.5389 | 0.4862 | 0.9713 | 1.336 | 0.065 | 8.31E-06 | IMP |
| **2** | rs2528592 | 159529272 | PKP4 | C | G | 0.5391 | 0.4864 | 0.971 | 1.3359 | 0.065 | 8.43E-06 | IMP |
| **2** | rs2711034 | 159530651 | PKP4 | A | T | 0.5386 | 0.4858 | 0.9652 | 1.3379 | 0.0652 | 8.02E-06 | IMP |
| **3** | chr3_77677315_D | 77677315 |  | I2 | D | 0.8496 | 0.9014 | 0.9652 | 0.6434 | 0.0991 | 8.57E-06 | IMP |
| **3** | rs283386 | 115415834 | GAP43 | A | G | 0.858 | 0.8958 | 0.7665 | 0.6098 | 0.1104 | 7.42E-06 | IMP |
| **4** | rs57800857 | 140863365 | MAML3 | A | C | 0.6855 | 0.6365 | 0.9212 | 1.3684 | 0.0707 | 9.16E-06 | IMP |
| **4** | rs35319653 | 140903155 | MAML3 | T | C | 0.3002 | 0.3514 | 0.9691 | 0.7328 | 0.0698 | 8.37E-06 | IMP |
| **4** | chr4_140905595_I | 140905595 | MAML3 | D | I3 | 0.6984 | 0.6467 | 0.9682 | 1.3655 | 0.0697 | 7.86E-06 | IMP |
| **4** | rs12505942 | 140906390 | MAML3 | T | C | 0.6996 | 0.6475 | 0.968 | 1.369 | 0.0698 | 6.84E-06 | IMP |
| **4** | rs13136239 | 140908755 | MAML3 | A | G | 0.3012 | 0.3534 | 0.9664 | 0.7293 | 0.0698 | 6.14E-06 | IMP |
| **4** | chr4_140909205_I | 140909205 | MAML3 | D | I9 | 0.686 | 0.6321 | 0.9323 | 1.3646 | 0.0703 | 9.75E-06 | IMP |
| **4** | rs6823515 | 140925403 | MAML3 | C | G | 0.3265 | 0.3825 | 0.9602 | 0.7372 | 0.0686 | 8.85E-06 | IMP |
| **5** | rs34416917 | 36406618 |  | A | G | 0.1156 | 0.1561 | 0.8749 | 0.6316 | 0.1026 | 7.49E-06 | IMP |
| **5** | rs35571570 | 36410724 |  | T | C | 0.1979 | 0.2522 | 0.9345 | 0.6981 | 0.0808 | 8.61E-06 | IMP |
| **5** | rs10512650 | 36419793 |  | T | C | 0.1892 | 0.2459 | 0.943 | 0.6943 | 0.0816 | 7.89E-06 | IMP |
| **6** | rs2145737 | 123879922 | TRDN | T | C | 0.6008 | 0.5422 | 0.9379 | 1.3526 | 0.0671 | 6.85E-06 | IMP |
| **6** | rs9365376 | 158198680 | SNX9 | T | C | 0.0693 | 0.1009 | 0.8188 | 0.5493 | 0.133 | 6.67E-06 | IMP |
| **6** | rs6922614 | 158206364 | SNX9 | T | C | 0.9205 | 0.888 | 0.8136 | 1.7794 | 0.1255 | 4.41E-06 | IMP |
| **7** | rs6975373 | 4467479 |  | T | C | 0.1056 | 0.0793 | 0.8489 | 1.7113 | 0.1186 | 5.95E-06 | IMP |
| **7** | rs75047832 | 4470076 |  | C | G | 0.1081 | 0.0814 | 0.8539 | 1.6776 | 0.1171 | 9.97E-06 | IMP |
| **7** | rs12530503 | 4493125 |  | A | C | 0.9241 | 0.9495 | 0.9481 | 0.5499 | 0.1344 | 8.58E-06 | IMP |
| **7** | rs114497090 | 19217524 |  | A | G | 0.0151 | 0.005 | 0.815 | 6.1683 | 0.4103 | 9.25E-06 | IMP |
| **7** | chr7_27669577_D | 27669577 |  | I5 | D | 0.3572 | 0.2951 | 0.9038 | 1.3789 | 0.0718 | 7.68E-06 | IMP |
| **7** | chr7_76213662_D | 76213662 |  | D | I4 | 0.229 | 0.189 | 0.8719 | 1.4694 | 0.084 | 4.63E-06 | IMP |
| **8** | rs11784341 | 96957730 | RP11-31K23.1 | T | C | 0.0245 | 0.0118 | 0.6765 | 4.2643 | 0.3038 | 1.81E-06 | IMP |
| **8** | rs140121824 | 96958935 | RP11-31K23.1 | A | C | 0.0245 | 0.0118 | 0.6765 | 4.2643 | 0.3038 | 1.81E-06 | IMP |
| **8** | rs150918060 | 96966136 |  | A | T | 0.0243 | 0.0119 | 0.6873 | 4.0694 | 0.2994 | 2.77E-06 | IMP |
| **8** | rs149724328 | 96977529 |  | A | G | 0.0239 | 0.012 | 0.7081 | 3.7974 | 0.293 | 5.27E-06 | IMP |
| **8** | rs183820757 | 96983770 |  | T | C | 0.0242 | 0.0121 | 0.7254 | 3.5986 | 0.2882 | 8.85E-06 | IMP |
| **9** | rs7859734 | 89562354 | GAS1 | T | C | 0.3234 | 0.3899 | 0.9284 | 0.7309 | 0.0699 | 7.34E-06 | IMP |
| **10** | rs184224980 | 12665138 | CAMK1D | T | C | 0.7877 | 0.8293 | 0.6115 | 0.6174 | 0.1039 | 3.44E-06 | IMP |
| **10** | rs12768957 | 82187293 | FAM213A | A | G | 0.1559 | 0.1232 | 0.9723 | 1.5119 | 0.0928 | 8.44E-06 | IMP |
| **10** | rs7074356 | 82191334 | FAM213A | A | G | 0.1586 | 0.1251 | 0.9643 | 1.5278 | 0.0926 | 4.75E-06 | IMP |
| **10** | rs35828718 | 82193877 |  | A | G | 0.156 | 0.1237 | 0.9611 | 1.5096 | 0.0932 | 9.98E-06 | IMP |
| **12** | rs113507694 | 7866609 | DPPA3 | A | G | 0.9427 | 0.9654 | 0.591 | 0.3524 | 0.2006 | 2.01E-07 | IMP |
| **12** | chr12_7867117_I | 7867117 | DPPA3 | I2 | D | 0.1707 | 0.1402 | 0.6188 | 1.6577 | 0.111 | 5.27E-06 | IMP |
| **12** | rs144017301 | 78915948 | RP11-171L9.1 | T | C | 0.8577 | 0.8111 | 0.9081 | 1.5204 | 0.0918 | 5.03E-06 | IMP |
| **12** | rs146457125 | 78916053 | RP11-171L9.1 | A | T | 0.8575 | 0.8107 | 0.9091 | 1.5209 | 0.0917 | 4.84E-06 | IMP |
| **12** | rs11110253 | 78918441 | RP11-171L9.1 | A | G | 0.8705 | 0.8255 | 0.8742 | 1.5641 | 0.0973 | 4.32E-06 | IMP |
| **12** | rs11110304 | 78928494 |  | T | C | 0.8494 | 0.8049 | 0.9059 | 1.4918 | 0.09 | 8.71E-06 | IMP |
| **13** | rs675828 | 103350733 | METTL21C | A | G | 0.4471 | 0.5172 | 0.9842 | 0.7496 | 0.0644 | 7.63E-06 | IMP |
| **13** | rs9520569 | 108402551 | FAM155A | T | C | 0.7808 | 0.7353 | 0.8278 | 1.4449 | 0.0824 | 7.86E-06 | IMP |
| **14** | rs187058036 | 87892807 | RP11-594C13.1 | T | C | 0.9611 | 0.9766 | 0.7328 | 0.3808 | 0.2159 | 7.74E-06 | IMP |
| **17** | rs76695126 | 9426724 | STX8 | T | C | 0.486 | 0.44 | 0.9092 | 1.3671 | 0.0674 | 3.47E-06 | IMP |
| **17** | rs1114532 | 9434564 | STX8 | T | C | 0.5205 | 0.565 | 0.9992 | 0.7517 | 0.0642 | 8.83E-06 | GT |
| **17** | chr17_9437507_D | 9437507 | STX8 | D | I3 | 0.4379 | 0.4772 | 0.804 | 0.7232 | 0.0719 | 6.54E-06 | IMP |
| **17** | rs4791850 | 9439561 | STX8 | T | C | 0.5222 | 0.5665 | 0.9862 | 0.7511 | 0.0647 | 9.57E-06 | IMP |
| **17** | rs1985762 | 48404076 |  | T | C | 0.1225 | 0.0832 | 0.814 | 1.6842 | 0.1164 | 7.56E-06 | IMP |
| **19** | rs12327596 | 54192903 | MIR526B | A | G | 0.5877 | 0.6468 | 0.9698 | 0.7412 | 0.0666 | 6.90E-06 | IMP |
| **19** | rs8104156 | 54193170 | MIR526B | T | G | 0.3995 | 0.3394 | 0.9527 | 1.3601 | 0.0677 | 5.47E-06 | IMP |
| **19** | rs1978714 | 54194677 | MIR526B | T | C | 0.5168 | 0.5755 | 0.9689 | 0.7479 | 0.0653 | 8.52E-06 | IMP |
| **19** | rs7248039 | 54196520 | MIR525 | A | G | 0.5185 | 0.577 | 0.9765 | 0.7495 | 0.065 | 9.32E-06 | IMP |
| **19** | rs7251482 | 54196955 | MIR525 | A | G | 0.5197 | 0.5789 | 0.9734 | 0.747 | 0.0652 | 7.65E-06 | IMP |
| **19** | rs10413288 | 54197633 | MIR525 | A | G | 0.5175 | 0.5767 | 0.9793 | 0.7481 | 0.0649 | 7.88E-06 | GT |
| **19** | rs8100449 | 54199017 | MIR525 | T | C | 0.5362 | 0.5955 | 0.94 | 0.7405 | 0.0665 | 6.25E-06 | IMP |
| **19** | rs8099860 | 54199251 | MIR525 | T | C | 0.4812 | 0.4218 | 0.9741 | 1.3388 | 0.0651 | 7.51E-06 | IMP |
| **19** | rs1989486 | 54200619 | MIR525 | A | G | 0.477 | 0.4181 | 0.9616 | 1.3469 | 0.0657 | 5.82E-06 | IMP |
| **19** | rs10417113 | 54201088 | MIR525 | T | C | 0.5184 | 0.5773 | 0.9732 | 0.7467 | 0.0652 | 7.39E-06 | IMP |
| **19** | rs10417538 | 54201286 | MIR525 | T | C | 0.5183 | 0.5773 | 0.9731 | 0.7464 | 0.0652 | 7.24E-06 | IMP |
| **19** | chr19_54202088_I | 54202088 | RPS9 | I5 | D | 0.4321 | 0.3786 | 0.836 | 1.3694 | 0.071 | 9.61E-06 | IMP |

## Supplementary Table 3: Overview of Gene Set Excytosis (GO: 0006887)

SNP = single nucleotide polymorphism; BP = Base pair position; CHR = chromosome

| *Significant Genes* | *SNP* | *BP* | *P-Value* | *CHR* | *Gene Start* | *Gene End* |
| --- | --- | --- | --- | --- | --- | --- |
| YKT6 | rs1004558 | 44240407 | 7,08E-05 | 7 | 44240567 | 44253893 |
| NLGN1 | rs9821957 | 173858380 | 4,30E-04 | 3 | 173114074 | 174004434 |
| LIN7A | rs17662201 | 81220111 | 6,04E-04 | 12 | 81186299 | 81331704 |
| RABEPK | rs393721 | 127985775 | 0,0045 | 9 | 127962821 | 127996437 |
| RIMS1 | rs6931045 | 72631747 | 0,0069 | 6 | 72596406 | 73112845 |
| SYT1 | rs10861133 | 79294033 | 0,0111 | 12 | 79257773 | 79845788 |
| PKDREJ | rs41302599 | 46657308 | 0,0126 | 22 | 46651560 | 46659219 |
| CADPS | rs11708441 | 62548081 | 0,0146 | 3 | 62384022 | 62861054 |
| AKAP3 | rs2074866 | 12680454 | 0,0151 | 7 | 4724674 | 4758213 |
| SCIN | rs55903142 | 160514 | 0,0161 | 17 | 12610203 | 12693228 |
| RPH3AL | rs1990313 | 4736086 | 0,0190 | 12 | 62293 | 236045 |
| CPLX1 | rs6816868 | 779670 | 0,0253 | 4 | 778745 | 819986 |
| CCL8 | rs8075846 | 32632483 | 0,0367 | 17 | 32646055 | 32648421 |
| SEPT05 | rs1557627 | 19677207 | 0,0380 | 22 | 19701987 | 19712295 |
| RAB26 | rs36232 | 2199788 | 0,0397 | 16 | 2190804 | 2204166 |
|  |  |  |  |  |  |  |
| *Non-Significant Genes* |  |  |  |  |  |  |
| SCRN1 | rs1049394 | 29960804 | 0,0554 | 7 | 29959719 | 30029905 |
| CPLX2 | rs1544923 | 175268492 | 0,0563 | 5 | 175223313 | 175311023 |
| ARFGEF2 | rs1997850 | 47526360 | 0,1248 | 20 | 47538427 | 47653230 |
| CCL5 | rs1065341 | 34198593 | 0,1940 | 17 | 34198495 | 34207797 |
| ARFGEF1 | rs10957384 | 68219564 | 0,2926 | 8 | 68085747 | 68255912 |
| VAMP3 | rs2071987 | 7834026 | 0,4460 | 1 | 7831329 | 7841492 |
| CCL3 | rs9972960 | 34420079 | 0,5845 | 17 | 34415602 | 34417515 |
|  |  |  |  |  |  |  |
|  |  | | | | | |
| *Genes without mapped variants* |  | | | | | |
| SYTL4 | No Variant | | | | | |
| VTI1B | No Variant | | | | | |
| SYTL4 | No Variant | | | | | |

## Supplementary Table 4: Association of polygenic risk scores for Bipolar Disorder (BIP), Schizophrenia (SCZ), and Major Depressive Disorder (MDD) with Borderline Personality Disorder case control status

R² = Nagelkerke’s R², N SNPs = Number of included SNPs

| *P-*value threshold | *BIP* | | | *SCZ* | |  | *MDD* | | |
| --- | --- | --- | --- | --- | --- | --- | --- | --- | --- |
|  | *N SNPs* | *R²* | *P* | *N SNPs* | *R²* | *P* | *N SNPs* | *R²* | *P* |
| 5*10^-8^ | 17 | 6.09E-05 | 0.74481 | 108 | 0.00493 | 0.00337 | 0 | - | - |
| 0.000001 | 73 | 0.00031 | 0.46040 | 246 | 0.01009 | 2.70E-05 | 5 | 1.49E-05 | 0.87201 |
| 0.0001 | 556 | 0.00310 | 0.02005 | 1,278 | 0.02211 | 4.78E-10 | 125 | 0.00012 | 0.65288 |
| 0.001 | 2,180 | 0.00497 | 0.00324 | 3,419 | 0.02993 | 4.04E-13 | 784 | 0.00468 | 0.00431 |
| 0.01 | 9,408 | 0.00670 | 0.00063 | 10,649 | 0.02929 | 7.23E-13 | 5,010 | 0.01211 | 4.20E-06 |
| 0.05 | 27,346 | 0.00691 | 0.00051 | 24,741 | 0.02714 | 5.04E-12 | 16,687 | 0.02115 | 1.14E-09 |
| 0.1 | 43,365 | 0.00731 | 0.00036 | 35,710 | 0.02757 | 3.40E-12 | 27,188 | 0.01736 | 3.52E-08 |
| 0.2 | 67,753 | 0.00856 | 0.00011 | 51,621 | 0.02765 | 3.18E-12 | 43,315 | 0.01841 | 1.36E-08 |
| 0.5 | 116,624 | 0.00778 | 0.00023 | 80,828 | 0.02993 | 4.03E-13 | 73,493 | 0.01515 | 2.64E-07 |
| 1 | 154,455 | 0.00670 | 0.00063 | 101,718 | 0.03062 | 2.17E-13 | 94,078 | 0.01511 | 2.73E-07 |

# Consortium Memberships

## Bipolar Disorders Working Group of the Psychiatric Genomics Consortium (Bipolar disorder)

Devin Absher, Rolf Adolfsson, Ingrid Agartz, Huda Akil, Martin Alda, Ney Alliey-Rodriguez, Ole A Andreassen, Adebayo Anjorin, Lena Backlund, Judith A Badner, Jack D Barchas, Nicholas J Bass, Michael Bauer, Bernhard T Baune, Frank Bellivier, Sarah E Bergen, Wade Berrettini, Andrew Bethell, Joanna M Biernacka, Douglas Blackwood, Cinnamon S Bloss, Michael Boehnke, Marco P Boks, Gerome Breen, René Breuer, William E Bunney, Margit Burmeister, William Byerley, Pablo Cervantes, Kim Chambert, Sven Cichon, David A Collier, Aiden Corvin, William Coryell, Nick Craddock, David W Craig, Cristiana Cruceanu, David Curtis, Piotr M Czerski, Anders Dale, Franziska Degenhardt, Jurgen Del‐Favero, Arianna Di Florio, Srdjan Djurovic, Amanda Dobbyn, Howard J Edenberg, Amanda Elkin, Torbjørn Elvsåshagen, Bruno Etain, Chun C Fan, Anne Farmer, Manuel Ferreira, Nicol Ferrier, Sascha Fischer, Matthew Flickinger, Tatiana Foroud, Andreas J Forstner, Liz Forty, Josef Frank, Christine Fraser, Nelson Freimer, Louise Frisén, Mark A Frye, Janice Fullerton, Elliot S Gershon, Michael Gill, Scott Gordon, Katherine Gordon-Smith, Elaine K Green, Tiffany A Greenwood, Maria Grigoroiu-Serbanescu, Maria Grigoroiu‐Serbanescu, Detelina Grozeva, Weihua Guan, Hugh Gurling, Jose Guzman-Parra, Marian L Hamshere, Joanna Hauser, Martin Hautzinger, Stefan Herms, Maria Hipolito, Per Hoffmann, Peter A Holmans, Laura Huckins, Christina Hultman, Stéphane Jamain, Ian R Jones, Lisa Jones, Anders Juréus, René Kahn, Radhika Kandaswamy, Robert Karlsson, John R Kelsoe, James L Kennedy, George Kirov, Sarah Kittel-Schneider, Sarah Knott, Manolis Kogevinas, Daniel L Koller, Ralph Kupka, Mikael Landén, Niklas Langstrom, Mark Lathrop, Jacob Lawrence, William B Lawson, Markus Leber, Marion Leboyer, Shawn Levy, Jun Li, Qingqin Li, Paul Lichtenstein, Jolanta Lissowska, Chunyu Liu, Falk W Lohoff, Susanne Lucae, Anna Maaser, Pamela B Mahon, Wolfgang Maier, Ulrik Malt, Nicholas Martin, Manuel Mattheisen, Keith Matthews, Morten Mattingsdal, Fermin Mayoral-Cleries, Susan McElroy, Kevin McGhee, Peter McGuffin, Melvin G McInnis, Andrew McIntosh, James D McKay, Alan W McLean, Francis J McMahon, Andrew McQuillin, Sarah Medland, Ingrid Melle, Fan Meng, Vihra Milanova, Philip B Mitchell, Grant W Montgomery, Jennifer Moran, Gunnar Morken, Derek Morris, Valentina Moskvina, Thomas W Mühleisen, Walter J Muir, Bertram Müller-Myhsok, Richard M Myers, Caroline M Nievergelt, Ivan Nikolov, Vishwajit Nimgaonkar, Annelie Nordin Adolfsson, Markus M Nöthen, John I Nurnberger, Evaristus A Nwulia, Michael C O'Donovan, Colm O'Dushlaine, Ketil J Oedegaard, Loes Olde Loohuis, Roel Ophoff, Anil Ori, Lilijana Oruc, Urban Osby, Michael J Owen, Sara Paciga, Carlos Pato, Roy Perlis, Amy Perry, Andrea Pfennig, Benjamin S Pickard, James B Potash, Peter Propping, Shaun Purcell, Emma Quinn, Soumya Raychaudhuri, Eline Regeer, Andreas Reif, Céline S Reinbold, John Rice, Marcella Rietschel, Fabio Rivas, Guy A Rouleau, Douglas Ruderfer, Martin Schalling, Alan F Schatzberg, William A Scheftner, Peter R Schofield, Nicholas J Schork, Thomas G Schulze, Johannes Schumacher, Markus Schwarz, Ed Scolnick, Laura J Scott, Paul D Shilling, Pamela Sklar, Erin N Smith, Jordan Smoller, Annet Spijker, David St. Clair, Eli Stahl, Michael Steffens, Eystein Stordal, John Strauss, Fabian Streit, Jana Strohmaier, Patrick Sullivan, Szabolcs Szelinger, Pippa Thomson, Robert C Thompson, Jens Treutlein, Gustavo Turecki, Arne Vaaler, John B Vincent, Yunpeng Wang, Stanley J Watson, Thomas F Wienker, Richard Williamson, Ashley Winslow, Stephanie H Witt, Adam Wright, Hualin Xi, Simon Xi, Wei Xu, Allan H Young, Peter P Zandi, Peng Zhang, Sebastian Zöllner

## Major Depressive Disorder Working Group of the Psychiatric Genomics Consortium (Major depressive disorder)

Stephan Ripke, Manuel Mattheisen, Abdel Abdellaoui, Mark J Adams, Esben Agerbo, Tracy M Air, Till F M Andlauer, Silviu-Alin Bacanu, Marie Bækvad-Hansen, Aartjan T F Beekman, David A Bennett, Klaus Berger, Tim B Bigdeli, Jonas Bybjerg-Grauholm, Enda M Byrne, Na Cai, Enrique Castelao, Toni-Kim Clarke, Jonathan R I Coleman, CONVERGE Consortium, Baptiste Couvy-Duchesne, Nick Craddock, Udo Dannlowski, Gareth Davies, Gail Davies, EJC de Geus, Philip De Jager, Ian J Deary, Franziska Degenhardt, Nese Direk, Erin C Dunn, Erik A Ehli, Thalia C Eley, Valentina Escott-Price, Tõnu Esko, Hilary K Finucane, Andreas J Forstner, Josef Frank, Michael Gill, Scott D Gordon, Jakob Grove, Lynsey S Hall, Thomas F Hansen, Christine Søholm Hansen, Thomas F Hansen, Caroline Hayward, Andrew C Heath, Anjali K Henders, Stefan Herms, IB Hickie, Per Hoffmann, Albert Hofman, Georg Homuth, Carsten Horn, Jouke- Jan Hottenga, David Hougaard, Hailiang Huang, Marcus Ising, Rick Jansen, Eric Jorgenson, Stefan Kloiber, James A Knowles, Warren W. Kretzschmar, Jesper Krogh, Zoltán Kutalik, Maren Lang, Glyn Lewis, Yihan Li, Donald J MacIntyre, Pamela AF Madden, Jonathan Marchine, Hamdi Mbarek, Patrick McGrath, Peter McGuffin, Sarah Elizabeth Medland, Divya Mehta, Andres Metspalu, Christel M Middeldorp, Evelin Mihailov, Yuri Milaneschi, Lili Milani, Grant W Montgomery, Sara Mostafavi, Niamh Mullins, Matthias Nauck, Bernard Ng, Merete Nordentoft, Dale R Nyholt, Michael C O'Donovan, Paul F O'Reilly, Hogni Oskarsson, Michael J Owen, Sara A Paciga, Carsten Bøcker Pedersen, Marianne Giørtz Pedersen, Nancy L Pedersen, Michele L Pergadia, Roseann E. Peterson, Erik Pettersson, Wouter J Peyrot, David J Porteous, Danielle Posthuma, James B Potash, Jorge A Quiroz, John P Rice, Brien P. Riley, Margarita Rivera, Douglas M. Ruderfer, Saira Saeed Mirza, Robert Schoevers, Thomas G Schulze, Ling Shen, Jianxin Shi, Engilbert Sigurdsson, Grant C B Sinnamon, Johannes H Smit, Daniel J Smith, Jordan W Smoller, Hreinn Stephansson, Stacy Steinberg, Fabian Streit, Jana Strohmaier, Katherine E Tansey, Alexander Teumer, Wesley Thompson, Pippa A Thomson, Thorgeir E Thorgeirsson, Jens Treutlein, Maciej Trzaskowski, André G Uitterlinden, Daniel Umbricht, Sandra Van der Auwera, Gerard van Grootheest, Albert M van Hemert, Alexander Viktorin, Henry Völzke, Yunpeng Wang, Bradley T. Webb, Myrna M Weissman, Jürgen Wellmann, Gonneke Willemsen, Stephanie H Witt, Hualin S Xi, Bernhard T Baune, Douglas H R Blackwood, Dorret I Boomsma, Anders D Børglum, Henriette N Buttenschøn, Sven Cichon, Enrico Domenici, Jonathan Flint, Hans J Grabe, Steven P Hamilton, Kenneth S Kendler, Qingqin S Li, Susanne Lucae, Patrik K Magnusson, Nicholas G Martin, Andrew M McIntosh, Ole Mors, Preben Bo Mortensen, Bertram Müller-Myhsok, Markus M Nöthen, Brenda WJH Penninx, Roy H Perlis, Martin Preisig, Marcella Rietschel, Catherine Schaefer, Jordan W Smoller, Kari Stephansson, Henning Tiemeier, Rudolf Uher, Thomas Werge, Ashley R Winslow, Gerome Breen, Douglas F Levinson, Cathryn M Lewis, Naomi R Wray, Patrick F Sullivan
